# 1671. Activity of Imipenem/Relebactam and Comparators Against Gram-Negative MDR and DTR Pathogens from Patients with Respiratory and Bloodstream Infections – SMART United States 2018-2020

**DOI:** 10.1093/ofid/ofac492.1301

**Published:** 2022-12-15

**Authors:** Sibylle Lob, Meredith Hackel, Fakhar Siddiqui, Karri A Bauer, Charles A DeRyke, Katherine Young, Mary Motyl, Daniel F Sahm

**Affiliations:** Merck & Co., Inc., Schaumburg, Illinois; IHMA, Schaumburg, Illinois; Merck & Co., Inc., Schaumburg, Illinois; Merck Research Laboratories, Kenilworth, New Jersey; Merck Research Laboratories, Kenilworth, New Jersey; Merck, Rahway, New Jersey; Merck, Rahway, New Jersey; IHMA, Schaumburg, Illinois

## Abstract

**Background:**

Gram-negative pathogens with multidrug resistance (MDR) or difficult-to-treat resistance (DTR) are increasingly common, resulting in limited treatment options. These resistant pathogens are often isolated from the respiratory tract or bloodstream and can result in significant patient morbidity and mortality. Imipenem/relebactam is a combination of imipenem with relebactam, a β-lactamase inhibitor of class A and C β-lactamases. We evaluated the activity of imipenem/relebactam and comparators (imipenem, meropenem, piperacillin/tazobactam, cefepime, ceftazidime, levofloxacin, and amikacin) against gram-negative MDR and DTR isolates that were collected from patients with lower respiratory tract (RTI) or bloodstream infections (BSI) in the United States (US).

**Methods:**

In 2018-2020, 24 clinical labs participated in the global SMART surveillance program in the US, and each collected up to 100 consecutive aerobic or facultative gram-negative pathogens per year from patients with RTI and 50 from BSI. MICs were determined using CLSI broth microdilution and interpreted with CLSI breakpoints.

**Results:**

MDR isolates were found among 13.5% of collected *P. aeruginosa* (n=2018), 11.9% of *E. coli* (n=1952), 13.1% of *K. pneumoniae* (n=1080), 24.1% of *E. cloacae* complex (n=460), 19.7% of *K. aerogenes* (n=304), and 6.1% of *S. marcescens* isolates (n=304); DTR isolates were found among 7.1%, 0.1%, 2.4%, 1.5%, 0%, and 0.8%, respectively. Imipenem/relebactam was active against 61% of MDR *P. aeruginosa*, 43-53 percentage points higher than the studied comparator β-lactams, and against 49% of DTR *P. aeruginosa*, which were nonsusceptible to all studied commonly used β-lactams and levofloxacin (Table). Imipenem/relebactam was also active against 87-100% of MDR Enterobacterales, including against 97% of MDR *K. pneumoniae*, 18-96 percentage points higher than the studied comparator β-lactams, and against 85% of DTR *K. pneumoniae*.

**Figure:**
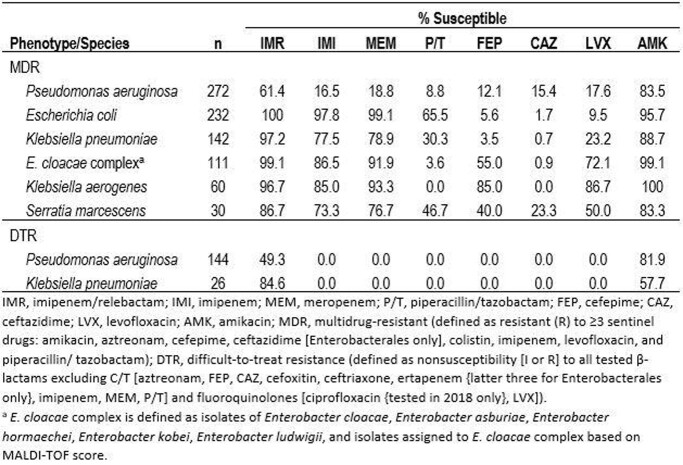

**Conclusion:**

Based on these *in vitro* data, imipenem/relebactam represents a promising treatment option in the US for patients with RTI or BSI caused by highly resistant gram-negative pathogens that pose substantial treatment challenges.

**Disclosures:**

**Fakhar Siddiqui, MD, MBA**, Merck & Co., Inc.: employee|Merck & Co., Inc.: Stocks/Bonds **Karri A Bauer, PharmD**, Merck & Co., Inc. Merck Research Laboratories: Stocks/Bonds **Charles A. DeRyke, PharmD**, Merck & Co., Inc. Merck Research Laboratories: Stocks/Bonds **Katherine Young, M.S.**, Merck & Co., Inc.: Stocks/Bonds.

